# Changes in Eating Habits among Displaced and Non-Displaced University Students

**DOI:** 10.3390/ijerph17155369

**Published:** 2020-07-25

**Authors:** Rita Bárbara, Cíntia Ferreira-Pêgo

**Affiliations:** 1 School of Sciences and Health Technologies, Universidade Lusófona de Humanidades e Tecnologias, Av. Campo Grande 376, 1749-024 Lisbon, Portugal; a21705573@alunos.ulht.pt; 2 CBIOS Lusófona’s Research Center for Biosciences and Health Technologies, Av. Campo Grande 376, 1749-024 Lisbon, Portugal

**Keywords:** university students, displaced students, Mediterranean diet adherence, food habit changes, Portuguese students

## Abstract

Nowadays the younger generations are moving their food habits from the traditional diet to a Western diet, which is low in fruits and vegetables and high in fat and sugary drinks. University students are a particularly vulnerable population once, with the entrance to university, they are subjected to new influences and responsibilities; in particular, those who live far from their parents’ houses are more predisposed to unhealthy eating habits. To assess the influence that admission to university has had on the frequency of intake of certain foods and meals as well as their adherence to the Mediterranean diet (MedDiet), self-administered questionnaires were applied. The sample included 97 Portuguese students, with an average age of 21 years, a normal weight, according to body mass index, and an average MedDiet adherence. Most of the individuals did not smoke and the majority did not drink coffee. It was also observed that displaced students consume fast food more frequently compared to the period before they start university. Fish ingestion decreased and coffee consumption increased, in the same group, after starting their university studies.

## 1. Introduction

Nowadays there seems to exist a pattern of unhealthy food choices among university students from different countries around the world, with low adherence to healthy dietary patterns, like the Mediterranean diet, unfavorable eating behaviors, and inadequate nutrient intake [1]. Especially the younger generations are moving away from the traditional dietary pattern and approaching the Western diet, characterized by low consumption of fruits and vegetables and high consumption of fast food and sugary drinks [2,3]. 

The university population can be divided into two categories, those who continue to live with their family and those who have had to move, as they attend universities away from home, commonly known as displaced students [4]. 

Most of the university students living far from home during this period experienced a transition from the family’s house to an independent adult life [4,5]. Thus, by leaving this family environment, in addition to the emotional and psychological changes to which they are subjected, university students still have the responsibility to decide their daily meals, which can predispose them to dietary errors and an unhealthy diet [5,6]. The establishment of eating habits during the university period is influenced by several factors, such as the stimulus of other students, the class schedule, alcohol consumption, and nightlife, or even the economic situation [7,8]. Therefore, university students are a vulnerable population, and displaced students are particularly prone to develop unfavorable eating habits [5]. In fact, in Italy, a study showed that changes in dietary habits happened mostly in displaced students and that displaced students not only consumed significantly more milk, packaged food, chips, beer, and spirits than their non-displaced peers but also reported a change in dietary habits since they started living alone [6]. Likewise, a study conducted in Greece found that university entrance did not lead to major dietary changes in non-displaced students, however, among the displaced ones, there was a decrease in the consumption of fresh fruit, vegetables, oily fish, seafood, legumes, and olive oil, and an increase of sugar, wine, alcohol, and fast food intake [5].

Unhealthy food choices are observed in several countries. In Finland, for example, a study showed a high adherence for unhealthy food groups, such as cake/cookies, snacks, fast food/canned food and sugar-sweetened beverages, and a moderate adherence for healthier food groups like fruit, vegetables, and cereals [1]. Furthermore, in Spain, the food choices of university students are characterized by a low intake of cereals and tubers, fruit, vegetables, legumes and nuts and olive oil, and a high content of processed meat, sweets, snacks, soft drinks, and pastries. Thereby, there is a greater consumption of foods present at the top of the food pyramid, which should be avoided, and consumption of vegetables, fruit, whole grains, nuts, and vegetables below the recommendations. Thus, a loss of adherence to the Mediterranean diet (MedDiet), both in Spain and other industrialized countries is observed [9,10].

For all these reasons, this study aims to assess changes in eating patterns and in MedDiet adherence of displaced and non-displaced university students, between before and after starting their university studies.

## 2. Materials and Methods 

### 2.1. Study Design and Population

The present study is a cross-sectional study designed to evaluate the influence that starting an independent life, outside the family home, has on eating patterns and adherence to the MedDiet of students who attend universities in the Lisbon region. Questionnaires were carried out through a digital platform and distributed through social networks to reach the largest number of university students. They were available between 30 October and 5 November 2019, and responses were obtained, randomly and anonymously, from 109 individuals. Exclusion criteria, such as being under 18 years old (4 individuals) or over 25 years old (5 individuals), not having Portuguese nationality (1 individual), not attending a university in the Lisbon region (1 individual) and having already completed the academic path (1 individual) was applied. Thus, 97 individuals remained in the study, with a mean age of 20.52 years. All participants gave their informed consent previous to study inclusion. The project was approved by the ethical committee (EC.ECTS/P02.19–20 December 2019) and conducted following the ethical standards as laid down in the 1964 Declaration of Helsinki and its later amendments or comparable ethical standards.

### 2.2. Dietary Habits and Adherence to the Mediterranean Diet

A questionnaire containing two parts was designed: the first category aimed to create a general characterization of the population and included sociodemographic questions, such as age, gender, anthropometric data, being a displaced student, academic area and degree, smoking and alcoholic habits, and supplementation use; and the second category aimed to understand whether there were changes in the eating pattern at the moment of admission to the university. 

With this proposal, a validated food frequency questionnaire was used to assess food consumption [11]. The present analysis was focused mainly on some particular foods and meals, such as meat, fish, eggs, dairy products, bread/pasta/rice, fruit, vegetables, legumes, fat, fast food (includes chips, pizza, etc.), ultra-processed food, water, coffee, and alcohol ingestion. The individuals answered the same questions twice, once related to their habits before university studies, and the other related to their present food habits as university students. Lastly, the differences between the two moments were calculated. A validated 14-item questionnaire was also applied to obtain the level of participants’ adherence to the MedDiet [12,13]. This questionnaire consisted of 14 questions, 12 about the frequency of food intake, and 2 questions on food intake habits considered characteristic of the Mediterranean diet (the main cooking fat used, and the meat preferably consumed). Each question was scored 0 (if the condition was not met) or 1 (if the condition was met). The final score ranged from 0 to 14 points, and then MedDiet was classified into three categories: “low adherence” (≤5 points), “average adherence” (between 6 and 9 points), and “high adherence” (≥10 points).

### 2.3. Statistical Analysis 

The data obtained were exported and processed in Microsoft Office Excel format, with the treatment consisting of categorizing the results. Statistical analysis was performed using SPSS Statistic software, version 26 (SPSS Inc., Chicago, IL, USA). Means and standard deviations (SD) were used as descriptive statistics for continuous variables, and percentages (%) and absolute values (*n*) were used to describe categorical variables. Differences in sociodemographic characteristics and food consumption, according to whether students were displaced or not, were tested with the Pearson χ^2^ test for categorical variables and ANOVA for continuous variables. For multiple comparisons, the Bonferroni post-hoc test was used. All statistical tests were two-tailed, and the significance level was set at *p* < 0.05.

## 3. Results

This study involved 97 Portuguese students attending universities in the Lisbon region, 64.90% of whom lived with their parents, and 35.10% were displaced. Table 1 shows the general characteristics of the population according to whether they are displaced or not. The study population has an average age of 20.52 years, a mean body mass index (BMI) of 22.39 kg/m^2^, and 75.30% of the individuals are female. There are statistically significant differences in the academic degree since it appears that the non-displaced students are predominantly pursuing graduation or an integrated master’s degree and most master’s students can be found in the displaced group. In addition, there is statistical significance in smoking habits, where there is a higher percentage of smokers and former smokers in the group of displaced students.

Table 2 describes the dietary characteristics of the population according to whether they are displaced students or not. There are statistically significant differences between the groups about coffee intake. There is a slight majority of non-consumers, with 55.70% of the total population reporting not consuming coffee, however, it should be noted that most coffee consumers belong to the group of displaced students. It is observed that the majority of the displaced students refer to coffee consumption, in contrast to the non-displaced, whose majority report not drinking coffee.

When asked about possible changes in their eating pattern, between the periods before and after arriving at the university, mostly we verify that there was a maintenance of the frequency of consumption, as can be seen in Table 3. This table shows statistically significant differences between groups in the consumption of fish, legumes, and fast food. It was found that the students who lived in their parents’ houses maintained the frequency of consumption of these foods. Displaced students, on the other hand, had a lower frequency of consumption of fish and higher consumption of fast food and legumes. 

The same analysis was also carried out by gender, with statistically significant differences being obtained only in the change in meat consumption (*p* = 0.001), showing that the majority of the female population consumes meat with less frequency, while the majority of male individuals consumes meat with increased his frequency (data not are shown). 

Differences between the answers of displaced and non-displaced students to the Mediterranean diet adherence questionnaire were also analyzed, as can be seen in Table 4. 

No statistically significant differences regarding MedDiet components or total adherence were found between the groups of displaced and non-displaced university students. This table also shows the classification of students’ adherence to the Mediterranean diet, noting that the majority of the studied population has an average adherence to this diet. There was no statistical significance between the groups either.

The same analysis was also carried out by gender and by academic area. In the first, there were statistically significant differences in the answers to following questions: Do you use olive oil as main culinary fat? (*p* = 0.039); How many fruit units (including natural fruit juices) do you consume per day? (*p* = 0.044); How many servings of red meat, hamburger, or meat products (ham, sausage, etc.) do you consume per day? (*p* = 0.013), showing that more female students scored on these questions than male students (data not shown). As for the academic area, there were statistically significant differences in the answers to questions: How much olive oil do you consume in a given day (including the oil used for frying, salads, out-of-house meals, etc.)? (*p* = 0.007) and How many servings of fish or shellfish do you consume per week? (*p* = 0.004), noting that students in health science area scored higher on these questions than their peers from other academic areas (data not shown).

## 4. Discussion

The results of the present study show that starting university studies can lead to changes in diet, especially in displaced students. These conclusions are consistent with a study by Papadaki et al. which suggested that leaving the family’s house predisposes students, responsible for meals for the first time, to change their eating habits [5]. 

There were statistically significant differences regarding academic degree, verifying that most of the population is studying for a graduation or an integrated master’s degree. Between groups, it appears that it is in the displaced group that most students obtaining a master’s degree are found, which can be explained in part because this group has an average age higher than the group of non-displaced students. However, another explanation could be the fact that students search for postgraduation studies closer to their interest area, and not their residence area, but there is no published scientific evidence on this topic. 

As for smoking habits, these are more present in the population of displaced students, both as active smokers and as former smokers. This is supported by a study by Beasley et al., in which it was found that this practice is more common in independent individuals when compared with those who lived with the family [8]. 

There were also statistically significant differences in the frequency of consumption of fish, legumes, and fast food, noting that in the group of non-displaced people the majority reported an equal frequency in the consumption of them all, unlike the displaced students who reported consuming fish less frequently and fast food and legumes with higher frequency. In the study by Lupi et al. it was found that students who lived with their parents consumed more fish than their displaced peers, however, as for the other foods mentioned above, namely legumes and fast food (includes chips, pizza, etc.), there were no statistically significant differences between displaced and non-displaced students [6]. This finding may suggest that living away from the family, and therefore from its influence, can lead the student to change his/her behavior, including making worse decisions about food, as suggested by Papadaki et al.; however, further studies are needed to confirm this hypothesis [5]. 

Regarding coffee consumption, it was found that the majority do not consume, and among those who do consume, most drink coffee once a day, contrasting with the results of Hadjimbei et al., where there was an average consumption of 2 to 3 times daily [14]. However, Lupi et al. observed an average consumption of tea/coffee close to 7 times a week, which is comparable with the results of the present study [6]. However, it should be taken into account that tea intake was not assessed by our researchers. Finally, it was found that the majority of students living with their parents do not consume coffee, unlike displaced students, most of whom drink at least one coffee daily. Lupi et al., in their study found the opposite; the students who reported consuming the most tea/coffee were those who lived with their parents, however, in this study, tea may be conditioning the conclusions we draw about coffee intake [6].

Regarding the dietary changes verified between genders, statistical significance was obtained only for meat consumption, with an equal or greater consumption of meat by male individuals and, on the contrary, an equal or lower consumption by female participants. The same was not verified in the study by Lupi et al. in which there were no statistically significant differences between genders, regarding the consumption of meat and poultry or meat products [6].

Regarding adherence to the MedDiet, there were no statistically significant differences between groups, either in the responses to the questionnaire or in the final classification obtained, and most of the university students showed an average adherence to the diet. This is supported by the study of Hadjimbei et al., even though their study used a questionnaire to evaluate Mediterranean diet adherence and assess the age group of children and adolescents, that was different from the one used in the present study [14]. On the other hand, a study carried out in Greece by Theodoridis et al. using the same questionnaire as the present study found that the majority of Greek students abandoned the Mediterranean diet, however, this study used different scores from ours to classify adherence to the diet, only classifying as “low adherence” and “high adherence”; we found it more pertinent to make a classification that included a middle ground [15]. When the differences in the responses to the questionnaire on adherence to the MedDiet by gender were analyzed, it was found that more female individuals had a greater preference for olive oil as a culinary fat, greater daily consumption of fruit and less daily consumption of red meat, hamburger or meat products when compared to their male counterparts. In a study by Cobo-Cuenca et al., it was observed with statistical significance that male students consumed more red meat than their female peers, which is consistent with our results. On the contrary, that same study found that male students consumed more fruit, with significant differences between genders. As for the preference for olive oil, there were no statistically significant differences between genders [16].

The same analysis was also carried out for the academic area, and it was found that students in the health area consumed more olive oil daily and more fish and shellfish weekly than students from other academic areas. A study from Péres-Gallardo et al. did not find significant differences between health and non-health students, regarding fish or fat consumption, however, in this study, the consumption of olive oil was not specified [17]. 

It is important to take into consideration that this study has some limitations. It should be noted that the questionnaire being self-administered may have led to its incorrect completion. Memory bias regarding the baseline and information bias due to social desirability cannot be excluded either. The cross-sectional design of the study is also considered a limitation since it does not allow to establish a cause–effect relation. In addition, the highest proportion of female individuals observed highlights the possibility that the sample is not representative of the male population. Finally, the small sample size can also be considered a limitation, however, it should be taken into consideration that this investigation is a pilot analysis of a cohort study. However, some strengths should be pointed out. This study is important once it can be taken as a starting point to identify causes and outline strategies to promote healthy habits of this population in particular, but also of society in the long-term, since by correcting students’ eating habits, these habits will remain throughout their lifetime. Thus, we consider this study an asset for the improvement of university students’ health and quality of life throughout their lives.

## 5. Conclusions

To the best of our knowledge, this is the first study to assess the changes that starting university studies induces in food choices of these students, displaced and non-displaced, in universities from the Lisbon region. Unlike their non-displaced peers, displaced students decreased their intake of fish and increased their consumption of fast food. Coffee consumption was also different between groups, with displaced students reporting consumption of at least one coffee per day.

In conclusion, university students are a vulnerable population group, especially the displaced ones, as it seems to be a trend towards unfavorable food choices. Thus, it is important to take measures to counter this trend, to promote long-term health in this fraction of the population.

## Figures and Tables

**Table 1:** General characteristics of the study population according to whether the university students are displaced from home or not.

	All Population (*n* = 97)	Not Displaced (*n* = 63)	Displaced (*n =* 34)	*p*-Value ^a^
**Age, years**	20.52 (2.46)	20.33 (2.44)	20.85 (2.50)	0.324
**Gender, % (*n*)**				
Male	24.70 (24)	25.40 (16)	23.50 (8)	0.839
Female	75.30 (73)	74.60 (47)	23.50 (8)	
**Height, m**	1.66 (0.08)	1.66 (0.08)	1.66 (0.09)	0.973
**Weight, kg**	61.66 (19.47)	63.22 (23.15)	58.77 (9.10)	0.285
**BMI, kg/m^2^**	22.39 (6.60)	22.95 (7.96)	21.35 (2.39)	0.257
**Academic Degree, % (*n*)**				
Graduation Degree	33.00 (32)	38.10 (24)	23.50 (8)	0.034
Integrated Master	52.60 (51)	54.00 (34)	50.00 (17)	
Master’s Degree	14.40 (14)	7.90 (5)	26.50 (9)	
**Academic Area, % (*n*)**				
Health	68.00 (66)	65.10 (41)	73.50 (25)	0.394
Others	32.00 (31)	34.90 (22)	26.50 (9)	
**Occupation, % (*n*)**				
Student	87.60 (85)	92.10 (58)	79.40 (27)	0.071
Working Student	12.40 (12)	7.90 (5)	20.60 (7)	
**Smoking Habits, % (*n*)**				
Non-Smoker	88.70 (86)	93.70 (59)	79.40 (27)	0.035
Smoker	5.20 (5)	4.80 (3)	5.90 (2)	
Former Smoker	6.20 (6)	1.60 (1)	14.70 (5)	
**Supplements use, % (*n*)**	15.50 (15)	17.50 (11)	11.80 (4)	0.459

Data expressed as means (SD) or percentages (*n*). Abbreviations: BMI, body mass index. ^a^ *p*-values for comparisons between groups were tested by Student’s t-test or Pearson χ^2^ as appropriate.

**Table 2:** Dietary characteristics of the study population according to whether the university students are displaced from home or not.

	All Population (*n* = 97)	Not Displaced (*n* = 63)	Displaced (*n* = 34)	*p*-Value ^a^
**Alcohol frequency of consumption, % (*n*)**	78.40 (76)	79.40 (50)	76.50 (26)	0.741
**Types of alcohol consumed, % (*n*)**				
Wine or Beer	24.70 (24)	25.40 (16)	23.50 (8)	0.969
White drinks	22.70 (22)	23.80 (15)	20.60 (7)	
None of the previous	30.90 (30)	30.20 (19)	32.40 (11)	
All of them	21.60 (21)	20.60 (13)	23.50 (8)	
**Fast Food Intake, % (*n*)**	89.70 (87)	88.90 (56)	91.20 (31)	0.724
**Fast Food Frequency, % (*n*)**				
≤1/month	71.10 (69)	76.20 (48)	61.80 (21)	0.135
>1/month	28.90 (28)	23.80 (15)	38.20 (13)	
**Coffee Intake, % (*n*)**	44.30 (43)	31.70 (20)	67.60 (23)	0.001
**Coffee Frequency, % (*n*)**				
Never	55.70 (54)	68.30 (43)	32.40 (11)	0.002
Once/day	25.80 (25)	20.60 (13)	35.30 (12)	
≥2/day	18.60 (18)	11.10 (7)	32.40 (11)	

Data expressed as percentages (*n*). ^a^ *p*-values for comparisons between groups were tested by Pearson χ^2^.

**Table 3:** Changes in the frequency of food intake, according to whether the university students are displaced from home or not.

	All Population (*n* = 97)	Not Displaced (*n* = 63)	Displaced (*n* = 34)	*p*-Value ^a^
**Meat, % (*n*)**				
<	29.90 (29)	27.00 (17)	35.30 (12)	0.355
=	56.70 (55)	61.90 (39)	47.10 (16)	
>	13.40 (13)	11.10 (7)	17.60 (6)	
**Fish, % (*n*)**				
<	27.80 (27)	17.50 (11)	47.10 (16)	0.001
=	53.60 (52)	66.70 (42)	29.40 (10)	
>	18.60 (18)	15.90 (10)	23.50 (8)	
**Eggs, % (*n*)**				
<	14.40 (14)	15.90 (10)	11.80 (4)	0.530
=	59.80 (58)	61.90 (39)	55.90 (19)	
>	25.80 (25)	22.20 (14)	32.40 (11)	
**Dairy Products, % (*n*)**				
<	25.80 (25)	23.80 (15)	29.40 (10)	0.482
=	66.00 (64)	69.80 (44)	58.80 (20)	
>	8.20 (8)	6.30 (4)	11.80 (4)	
**Bread, Pasta and Rice, % (*n*)**				
<	11.30 (11)	9.50 (6)	14.70(5)	0.305
=	66.00 (64)	71.40 (45)	55.90 (19)	
>	22.70 (22)	19.00 (12)	29.40 (10)	
**Fruit, % (*n*)**				
<	16.50 (16)	12.70 (8)	23.50 (8)	0.059
=	51.50 (50)	60.30 (38)	35.30 (12)	
>	32.00 (31)	27.00 (17)	41.20 (14)	
**Vegetables, % (*n*)**				
<	13.40 (13)	9.50 (6)	20.60 (7)	0.311
=	47.40 (46)	49.20 (31)	44.10 (15)	
>	39.20 (38)	41.30 (26)	35.30 (12)	
**Legumes, % (*n*)**				
<	18.60 (18)	11.10 (7)	32.40 (11)	0.002
=	55.70 (54)	68.30 (43)	32.40 (11)	
>	25.80 (25)	20.60 (13)	35.30 (12)	
**Fat, % (*n*)**				
<	20.60 (20)	19.00 (12)	23.50 (8)	0.678
=	78.40 (76)	79.40 (50)	76.50 (26)	
>	1.00 (1)	1.60 (1)	0.00 (0)	
**Fast Food, % (*n*)**				
<	30.90 (30)	36.50 (23)	20.60 (7)	0.027
=	41.20 (40)	44.40 (28)	35.30 (12)	
>	27.80 (27)	19.00 (12)	44.10 (15)	
**Ultra-Processed Products, % (*n*)**				
<	22.70 (22)	27.00 (17)	14.70 (5)	0.282
=	54.60 (53)	54.00 (34)	55.90 (19)	
>	22.70 (22)	19.00 (12)	29.40 (10)	
**Water, % (*n*)**				
<	9.30 (9)	7.90 (5)	11.80 (4)	0.206
=	40.20 (39)	34.90 (22)	50.00 (17)	
>	50.50 (49)	57.10 (36)	38.20 (13)	
**Coffee, % (*n*)**				
<	11.30 (11)	15.90 (10)	2.90 (1)	0.068
=	45.50 (44)	47.60 (30)	41.20 (14)	
>	43.30 (42)	36.50 (23)	55.90 (19)	
**Alcohol, % (n)**				
<	8.20 (8)	7.90 (5)	8.80 (3)	0.962
=	57.70 (56)	58.70 (37)	55.90 (19)	
>	34.00 (33)	33.30 (21)	35.30 (12)	
**Breakfast, % (*n*)**				
<	16.50 (16)	12.70 (8)	23.50 (8)	0.265
=	66.00 (64)	71.40 (45)	55.90 (19)	
>	17.50 (17)	15.90 (10)	20.60 (7)	
**No. of meals, % (*n*)**				
<	27.80 (27)	25.40 (16)	32.40 (11)	0.668
=	53.60 (52)	54.00 (34)	52.90 (18)	
>	18.60 (18)	20.60 (13)	14.70 (5)	

Data expressed as percentages (*n*). Abbreviations: <, decrease in frequency; =, maintain frequency; >, increase frequency. ^a^ *p*-values for comparisons between groups were tested by Pearson χ^2^.

**Table 4:** Mediterranean diet adherence according to whether the university students are displaced from home or not.

	All Population (*n* = 97)	Not Displaced (*n* = 63)	Displaced (*n* = 34)	*p*-Value ^a^
Adherence to MedDiet, % (*n*) *				
Low	5.20 (5)	4.80 (3)	5.90 (2)	0.898
Average	61.90 (60)	63.50 (40)	58.80 (20)	
High	33.00 (32)	21.70 (20)	35.30 (12)	
**MedDiet Questionnaire, % (*n*)**				
Olive oil as main culinary fat	92.80 (90)	95.20 (60)	88.20 (30)	0.203
≥4 tbsp of olive oil/day	35.10 (34)	38.10 (24)	29.40 (10)	0.392
≥2 vegetable servings/day	48.50 (47)	4.00 (29)	52.90 (18)	0.516
≥3 fruit units/day	38.10 (37)	42.90 (27)	29.40 (10)	0.193
≤1 serving of red meat or derivatives/day	62.90 (61)	61.90 (39)	64.70 (22)	0.785
≤1 serving of butter, margarine, cream/day	83.50 (81)	81.00 (51)	88.20 (30)	0.356
≤1 sweet or carbonated beverages/day	95.90 (93)	98.40 (62)	91.20 (31)	0.087
≥7 glasses of wine/week	2.10 (2)	1.60 (1)	2.90 (1)	0.654
≥3 servings of legumes/week.	86.60 (84)	88.90 (56)	82.40 (28)	0.367
≥3 servings of fish or shellfish/week	59.80 (58)	55.60 (35)	67.60 (23)	0.247
<3 servings commercial sweets or pastries/week	47.40 (46)	47.60 (30)	47.10 (16)	0.958
≥3 servings of nuts/week	35.10 (34)	34.90 (22)	35.30 (12)	0.971
Preferentially white meat instead of red meat	84.50 (82)	81.00 (51)	91.20 (31)	0.184
≥2 servings of sofrito/week	89.70 (87)	88.90 (56)	91.20 (31)	0.724

Data expressed as percentages (*n*). Abbreviations: MedDiet, Mediterranean Diet; * Low, 5 points or fewer; Average, between 6 and 9 points; High, 10 or more points. ^a^ *p*-values for comparisons between groups were tested by Pearson χ^2^.
